# Non-thrombotic Abnormalities on Lower Extremity Venous Duplex Ultrasound Examinations

**DOI:** 10.5811/westjem.2014.12.24170

**Published:** 2015-03-02

**Authors:** Srikar Adhikari, Wes Zeger

**Affiliations:** * University of Arizona Medical Center, Department of Emergency Medicine, Tucson, Arizona; † University of Nebraska Medical Center, Department of Emergency Medicine, Omaha, Nebraska

## Abstract

**Introduction:**

Emergency physician-performed compression ultrasonography focuses primarily on the evaluation of the proximal veins of the lower extremity in patients with suspected deep venous thrombosis (DVT). A detailed sonographic evaluation of lower extremity is not performed. The objective of this study was to determine the prevalence of non-thrombotic findings on comprehensive lower extremity venous duplex ultrasound (US) examinations performed on emergency department (ED) patients.

**Methods:**

We performed a retrospective six-year review of an academic ED’s records of adult patients who underwent a comprehensive lower extremity duplex venous US examination for the evaluation of DVT. The entire US report was thoroughly reviewed for non-thrombotic findings.

**Results:**

We detected non-thrombotic findings in 263 (11%, 95% CI [9.5–11.9%]) patients. Among the non-thrombotic findings, venous valvular incompetence (81, 30%) was the most frequent, followed by cyst/mass (41, 15%), lymphadenopathy (33, 12%), phlebitis (12, 4.5%), hematoma (8, 3%), cellulitis (1, 0.3%) and other (6, 2.2%).

**Conclusion:**

In our study, we detected a variety of non-thrombotic abnormalities on comprehensive lower extremity venous duplex US examinations performed on ED patients. Some of these abnormalities could be clinically significant and potentially be detected with point-of-care lower extremity US examinations if the symptomatic region is evaluated. In addition to assessment of the proximal veins for DVT, we recommend sonographic evaluation of the symptomatic area in the lower extremity when performing point-of-care ultrasound examinations to identify non-thrombotic abnormalities that may require immediate intervention or close follow up.

## INTRODUCTION

Deep venous thrombosis (DVT) affects approximately 250,000 individuals annually in the United States with an average incidence of one person per 1,000 population.^1^,^2^ Data from the National Ambulatory Medical Care Survey and National Hospital Ambulatory Medical Care Survey suggest that a majority of DVT diagnoses were made in the emergency department (ED) setting.^3^ Since the incidence of this disease is so high and progression from DVT to pulmonary embolism can result in significant morbidity and mortality, early detection and treatment of DVT is critical to improve patient outcomes. Duplex ultrasonography of the lower extremity has become the first-line diagnostic test to detect DVT, with a sensitivity of 91% to 96% and a specificity of 98% to 100%.^4^

In the recent years, ultrasound (US) equipment has become more compact, portable, and affordable, which has facilitated the rapid evolution of emergency ultrasonography.^5^ The use of point-of-care US by emergency physicians is increasing dramatically both for diagnostic purposes and procedural guidance. The accuracy and noninvasive characteristics of portable US make it an excellent tool for rapid diagnosis of serious and life-threatening conditions in the ED.^6^ Prior studies have shown that emergency physician-performed bedside US improves diagnostic accuracy, procedural safety and ED throughput.^7^–^9^

The accuracy of emergency physician-performed compression ultrasonography in the diagnosis of DVT has been extensively studied. In a recent systematic review and meta-analysis, the estimates for emergency physician-performed compression ultrasonography sensitivity and specificity for detecting DVT were 96.1% and 96.8% respectively.^10^ A simplified three-point or two-point compression technique is generally used for DVT evaluation by emergency physicians.^11^,^12^ Two-point compression (common femoral vein and popliteal vein) ultrasonography was found to be equivalent to whole-leg ultrasonography when used for the management of symptomatic patients with suspected DVT.^13^ Regardless of the technique used, emergency physician-performed ultrasonography focuses primarily on the evaluation of proximal veins of lower extremity. A detailed sonographic evaluation of the lower extremity is not performed. In contrast, whole-leg ultrasonography can detect conditions other than venous thrombosis that may be causing leg symptoms. It is unclear if emergency physicians should modify the lower extremity point-of-care US technique to assess for non-thrombotic abnormalities. To our knowledge, the clinical significance of failure to evaluate for non-thrombotic abnormalities with point-of-care compression ultrasonography has not been studied. It is important to understand the nature of the non-thrombotic abnormalities in order to assess the significance of such findings and determine the implications for point-of-care compression ultrasonography.

The objective of this study was to determine the prevalence of non-thrombotic findings on comprehensive lower extremity venous duplex US examinations performed in ED patients.

## METHODS

### Study Design

This was a retrospective review of ED patients who received a lower extremity venous duplex US examination over a six-year period. The institutional review board at our institution approved this study.

### Study Setting and Population

We conducted this study at an academic medical center with an annual ED census of approximately 45,000 patients. The ED has a residency training program and an active emergency sonography program. The primary investigators of this study were two emergency physicians with expertise in bedside US. We included in this study all adult patients who received a lower extremity venous duplex US examination for evaluation of DVT in the ED.

### Study Protocol

A retrospective review of adult (≥19 years) patients who presented to the ED with symptoms suspicious for DVT and received a comprehensive lower extremity venous US examination were included in this study. ED visits for the study period were extracted from the hospital electronic medical record system. We identified all ED patients who received a comprehensive lower extremity venous duplex ultrasound examination during the study period using current procedural terminology (CPT) code for lower extremity venous duplex US examination from our DVT research database.^14^ In the ED, clinical assessment was performed by emergency medicine residents and faculty. All subjects also underwent a single comprehensive lower extremity duplex venous US (B-mode and Doppler) for evaluation of DVT in the ED. The US examinations were performed by vascular surgery division sonographers and interpreted by board-certified vascular surgeons. The US protocol included both B-mode and Doppler color flow analysis of deep veins of lower extremity including calf veins. B-mode imaging of lower extremity veins without and with transducer compressions was performed for assessing venous patency at each of the following levels: common femoral vein, junction of the common femoral vein with the greater saphenous vein, femoral vein, deep femoral vein, popliteal vein, anterior tibial vein, posterior tibial vein and peroneal vein. Spectral Doppler waveforms of lower extremity veins were obtained showing variations with respiration and/or flow augmentation. Any vascular and nonvascular abnormalities, if detected were thoroughly assessed. We did not include point-of-care US data in this study due to inconsistencies in documentation and US image archiving.

Three physicians independently performed data extraction from medical records after a training session to standardize data collection strategies. The training session lasted approximately four hours. The data abstractors were not blinded to study objectives. A standardized data extraction form was used for data collection from medical records. Any discrepancies in the data extraction were resolved by discussion between the data abstractors. They reviewed medical records for final US reports. The entire US report was thoroughly reviewed for non-thrombotic findings. Each US report was initially reviewed by only one data abstractor. To assess the accuracy of data extraction, a second data abstractor then reviewed a randomly sampled 20% of US reports.

### Data Analysis

We used descriptive statistics to summarize the data using SAS software 9.1 (SAS Institute Inc., Cary, North Carolina, USA). Continuous data are presented as percent frequency of occurrence with 95% confidence intervals. Inter-observer agreement among data abstractors for the presence of non-thrombotic findings was assessed by kappa analysis.

## RESULTS

**Figure 1:**
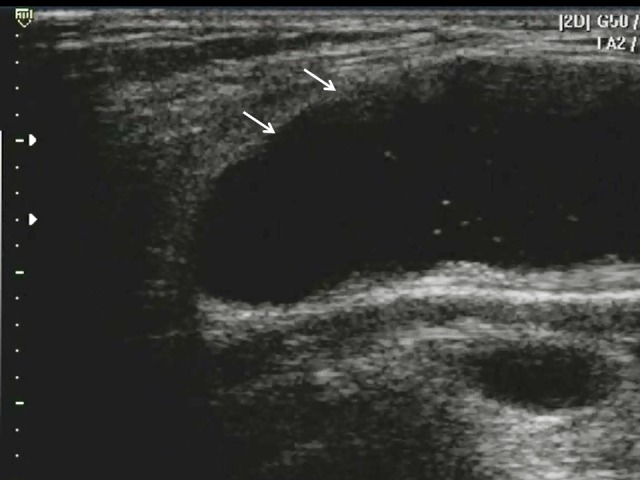
Baker’s cyst (arrows, anechoic distension of the semimembranosus-medial gastrocnemius bursa).

**Figure 2:**
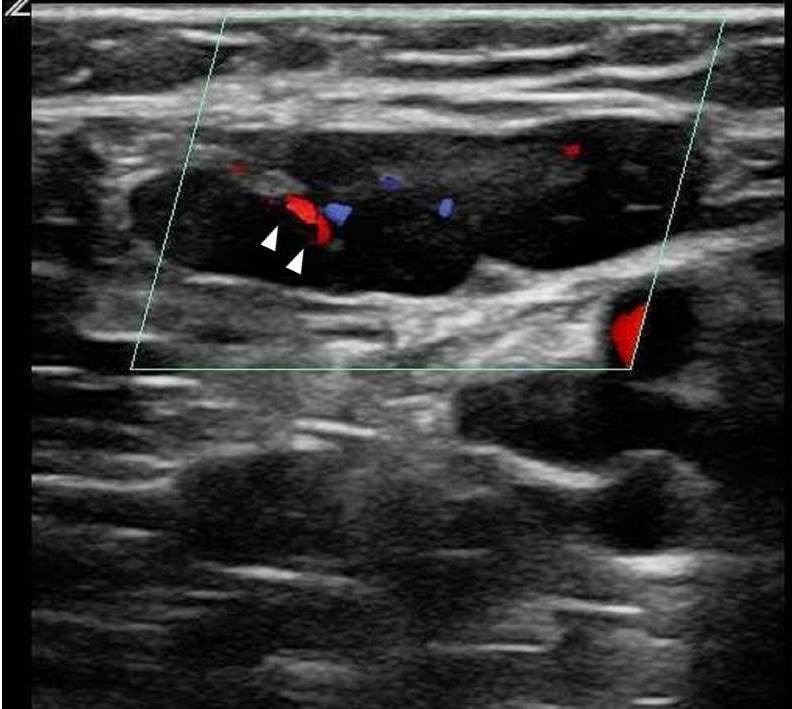
Enlarged inguinal lymph node (arrows) demonstrating flow in the hilar region with Color Doppler (arrowheads).

**Figure 3:**
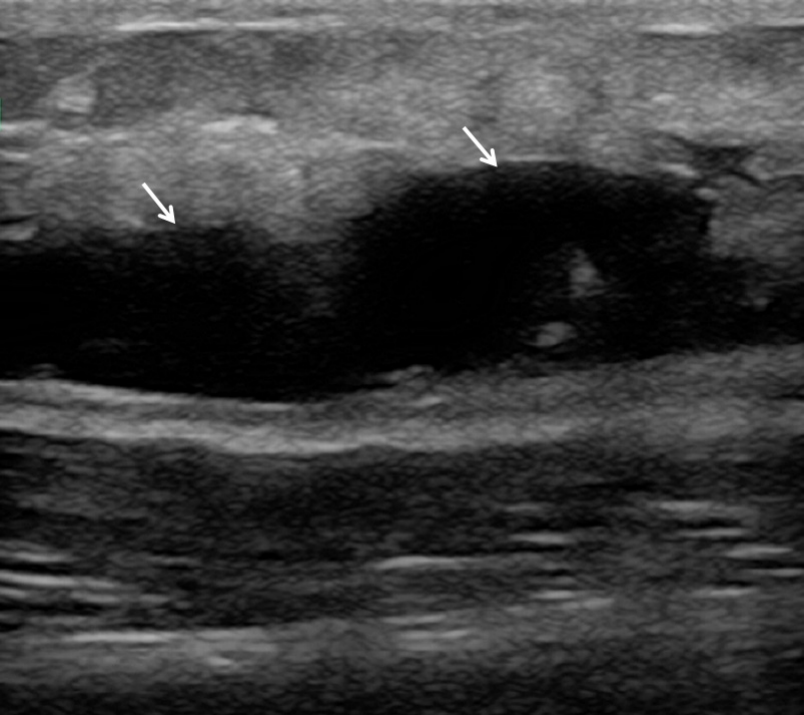
Liquefied hematoma (arrows, anechoic fluid collection).

**Figure 4:**
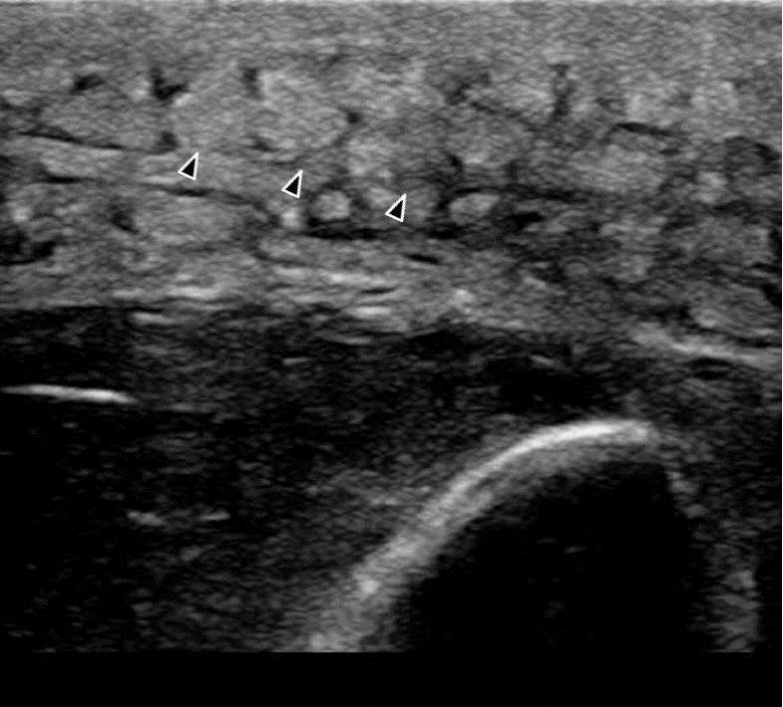
Cobblestone appearance (arrowheads) of subcutaneous tissues suggesting cellulitis.

A total of 2,390 lower extremity US reports were reviewed. Non-thrombotic findings were detected in 263 (11%, 95% CI [9.5–11.9%]) patients over the six-year period. Inter-observer agreement among chart reviewers for the presence of non-thrombotic findings was high (k=0.98). The different non-thrombotic findings found in lower extremity venous duplex US examinations are summarized in the Table (Figures 1–4 and Videos 1–4). Some patients had more than one non-thrombotic abnormality.

Among the non-thrombotic findings, venous valvular incompetence was the most frequent finding. In patients who had a mass or cyst, Baker’s cyst was found in 27 cases. Bilateral Baker’s cysts were found in three patients. One patient had a loculated Baker’s cyst. Four patients were found to have a mass in the calf. One patient had a fluid-filled mass in the distal left thigh with no vascular flow signals. Six patients (categorized as “other” in the table) had the following abnormalities: popliteal artery aneurysm with evidence of distal ischemia, bilateral popliteal artery aneurysms, subcutaneous fluid in calf, superficial femoral artery occlusion with monophasic posterior tibial artery waveforms, ankle effusion and absence of Doppler signals in the pedal arteries.

## DISCUSSION

Only a small proportion (15–25%) of patients with clinically suspected DVT have objective evidence of thrombosis when evaluated by ultrasonography.^15^ As a result, an alternative diagnosis is considered in a majority of patients. Few prior studies have explored alternate diagnoses in patients with suspected DVT who received a lower extremity US examination.^15^–^17^ In contrast to our study,^10^ Cate-Hoek et al. studied patients with suspected DVT in the primary care setting. The alternative diagnoses were based on clinical evaluation. The most common alternative diagnoses were muscle rupture, chronic venous insufficiency, erysipelas/cellulitis and superficial venous thrombosis. Lower extremity ultrasonography did not improve the diagnostic yield of alternative diagnoses.

Generally, incidental findings detected on lower extremity US do not alter management of ED patients. Most of these abnormalities do not require urgent treatment, admission or further evaluation during the ED visit. However, conditions such as abscess or hematoma require immediate attention in the ED. These diagnoses are not always clear clinically, especially in the early stages of the disease. Clinical criteria and laboratory data alone are not always helpful to detect the underlying pathology. If not diagnosed and treated in a timely fashion, these conditions may lead to serious complications in patients taking anticoagulants or immunosuppressed patients. Sonographic evaluation of proximal veins alone may not be sufficient in all patients presenting to the ED with lower extremity symptoms. In addition to evaluation of the proximal veins for DVT, we recommend a quick US evaluation of the symptomatic area in the lower extremity to identify these abnormalities. Depending on the expertise of the physician sonologist, a follow-up radiology department US examination may be necessary to further evaluate the abnormalities detected on point-of-care US examinations. Scanning the symptomatic subcutaneous and musculoskeletal regions may aid the clinician in formulating the appropriate treatment and follow-up plans. By adopting this approach, emergency physicians can quickly identify conditions that require immediate therapy (incision and drainage for abscess, or anticoagulant dose adjustment in the presence of hematoma) from those that need less urgent intervention. This approach may also help determine the urgency of follow up and increase patient satisfaction. Currently, not all patients who undergo a point-of-care lower extremity venous US examination are instructed to obtain a follow-up comprehensive lower extremity venous US examination. Additionally, patient compliance with follow-up US examinations was found to be extremely low.^18^ Scanning the symptomatic region in addition to the assessment of proximal veins for DVT may help physicians determine who needs a follow-up US examination for non-thrombotic abnormalities.

## LIMITATIONS

Our study has several methodological limitations, which may limit the conclusions that can be reached. The most important limitation of this study is that we did not review patient outcomes. It would have been helpful to know the clinical outcomes of patients with non-thrombotic abnormalities in order to determine what proportion were clinically significant. However, based on the nature of the non-thrombotic abnormalities detected in this study, it is reasonable to assume that a significant proportion of our patients required further evaluation and some patients needed emergent treatment including operative intervention. Therefore, we recommend sonographic evaluation of the symptomatic region in the lower extremity while performing point-of-care lower extremity US examinations. The retrospective study design could have introduced selection bias. However, the database that was used to identify our subjects captured all lower extremity venous US examinations performed on ED patients. We were able to obtain final US reports on all consecutive patients who underwent lower extremity venous US examinations. Another limitation is that our study was conducted at a single tertiary care academic center, and results may not be generalizable to other settings. Our vascular surgery division’s US protocol includes a complete lower extremity evaluation including assessment of an incidental finding if detected. It is possible that US reports reviewed for data collection may have missed some incidental findings that were not routinely reported. Additionally, we did not include point-of-care compression US examination data in our analyses. In our study, only one patient was found to have cellulitis, which is more commonly found in patients who receive lower extremity US examinations for suspicion of DVT.^16^ We only analyzed US reports and did not examine the clinical course, additional diagnostic testing, treatment and resolution of symptoms in these patients.

## CONCLUSION

In our study, a variety of non-thrombotic abnormalities were detected on comprehensive lower extremity venous duplex US examinations performed on ED patients. Some of these abnormalities could be clinically significant and can potentially be detected with point-of-care lower extremity US examinations if the symptomatic region is evaluated. In addition to assessment of the proximal veins for DVT, we recommend sonographic evaluation of the symptomatic area in the lower extremity when performing point-of-care ultrasound examinations to identify non-thrombotic abnormalities which may require immediate intervention or close follow up.

## Figures and Tables

**Video 1:** Baker’s Cyst.

**Video 2:** Enlarged inguinal lymph node.

**Video 3:** Liquified hematoma in calf region.

**Video 4:** Cellulitis.

**Table:** Non-thrombotic findings detected in lower extremity venous duplex ultrasound examinations.

Non-thrombotic findings	N (%) [95% CI]
Venous valvular incompetence	81 (30%) [76.3% – 85.7%]
Cyst/mass	41 (15%) [35.1% – 46.9%]
Lymphadenopathy	33 (12%) [27.3% – 38.7%]
Phlebitis	12 (4.5%) [76.3% – 85.7%]
Hematoma	8 (3%) [8.1% – 15.9%]
Cellulitis	1 (0.3%) [−0.2% – 2.2%]
Other	6 (2.2%) [3.1% – 8.9%]
